# P-860. Association between palliative care consultation and use of broad-spectrum antibiotics in patients with advanced cancer during the end-of-life trajectory: a retrospective cohort study with nationwide linkage

**DOI:** 10.1093/ofid/ofaf695.1068

**Published:** 2026-01-11

**Authors:** Jeong-Han Kim, Jiwon Yu, Ye Sul Jeung, Shin Hye Yoo, Jin-ah Sim, Bhumsuk Keam

**Affiliations:** Ewha Woman University College of Medicine Mokdong Hospital, Department of Internal Medicine, Yangcheon-gu, Seoul-t'ukpyolsi, Republic of Korea; Seoul National University College of Medicine, 2Department of Biomedical Sciences, Jongno-gu, Seoul-t'ukpyolsi, Republic of Korea; Seoul National University Hospital, Center for Palliative Care and Clinical Ethics, Jongno-gu, Seoul-t'ukpyolsi, Republic of Korea; Seoul National University Hospital, Center for Palliative Care and Clinical Ethics, Jongno-gu, Seoul-t'ukpyolsi, Republic of Korea; Hallym University, Department of AI Convergene, Chencheon, Kangwon-do, Republic of Korea; Seoul National University Hospital, Department of Internal Medicine, Jongno-gu, Seoul-t'ukpyolsi, Republic of Korea

## Abstract

**Background:**

Despite increasing awareness of broad-spectrum antibiotic overuse in patients with advanced cancer at the end of life, prescription decision-making remains a challenging issue in this population. This study evaluated the impact of palliative care (PC) consultation on antibiotic prescribing patterns throughout the end-of-life trajectory.

**Figure 1.**
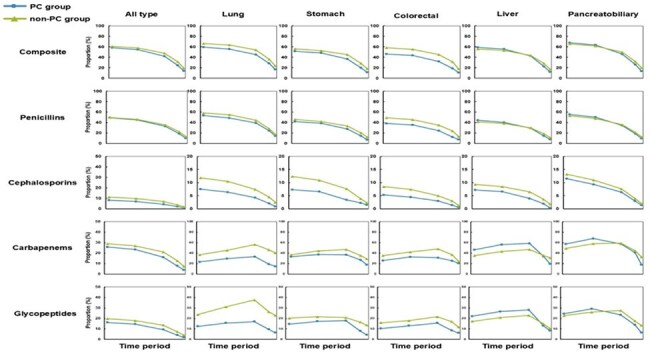
Broad-Spectrum Antibiotic Prescription Proportions by Cancer Type and Antibiotic Class Across End-of-Life Intervals (T1–T5).

**Figure 2.**
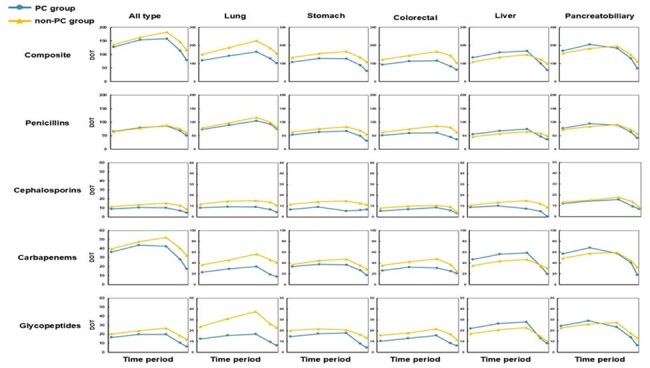
Days of Therapy (DOT) for Broad-Spectrum Antibiotics by Cancer Type and Antibiotic Class Across End-of-Life Intervals (T1–T5).

**Methods:**

This retrospective cohort study included adults (≥19 years) diagnosed with advance stage of lung, gastric, colorectal, liver, or pancreatobiliary cancer who received care at a tertiary hospital between 2018 and 2022 and had a confirmed date of death by June 23, 2023. Healthcare utilization data were obtained by linking institutional records with the National Health Insurance Service database. The end-of-life trajectory was divided into five intervals: T1 (6–3 months before death), T2 (3–1 month), T3 (1 month to 2 weeks), T4 (2–1 week), and T5 (last week before death). Broad-spectrum antibiotic use was compared between PC and non-PC groups using prescription proportion and days of therapy (DOT) per 1,000 patient-days, with baseline differences adjusted using 1:2 propensity score matching.

**Table 1.**
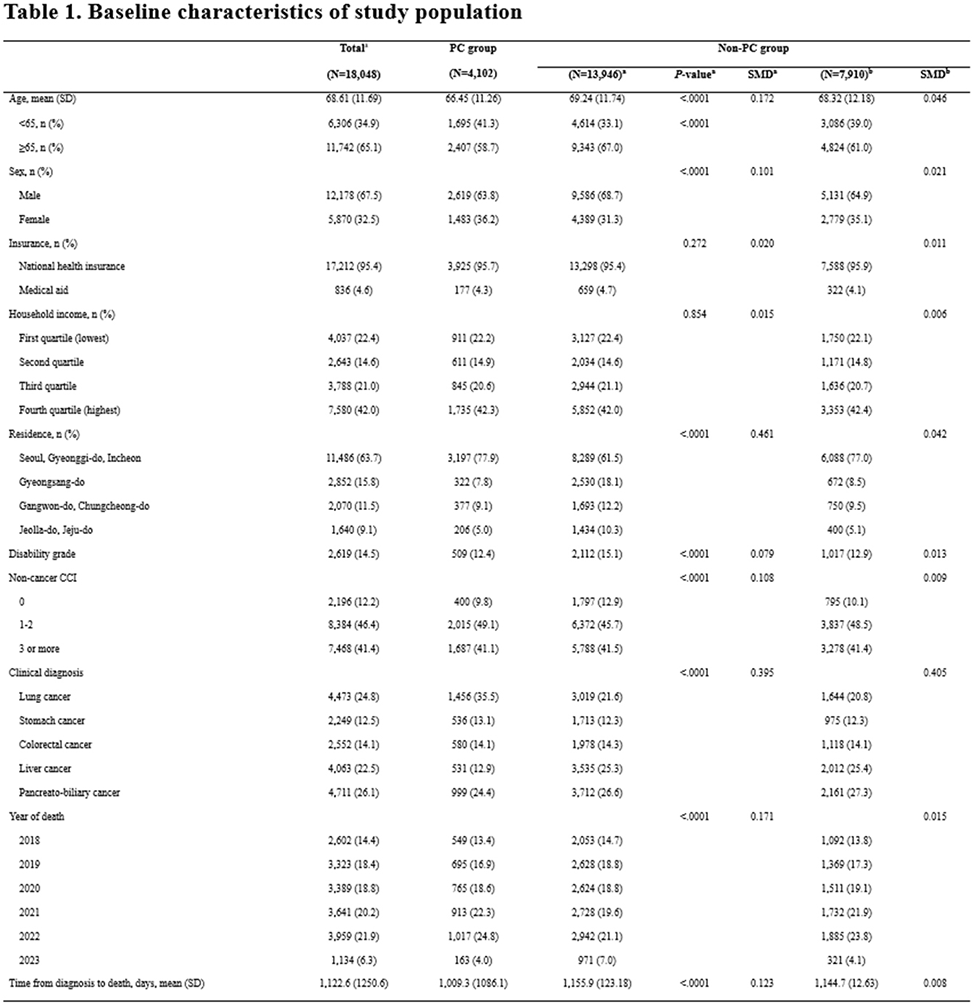
Baseline characteristics of study population

**Results:**

Among 18,048 patients included, 4,102 received PC consultation. After propensity score matching, 7,910 non-PC patients were retained for comparison. During the last six months of life, 70.6% of PC patients and 72.9% of non-PC patients received at least one prescription of broad-spectrum antibiotics, whereas DOT was 107.6 vs. 125.0 per 1,000 patient-days, respectively. The PC group consistently showed lower prescription proportions and DOT across all end-of-life intervals. Prescription proportions peaked in T1 (58.3% vs. 61.1%) and declined steadily over time to T5 (13.8% vs. 19.4%). In contrast, DOT was highest in T3 (158.8 vs. 182.6 per 1,000 patient-days) and declined to T5 (79.0 vs. 116.0 per 1,000 patient-days). This pattern was consistent across antibiotic classes. Reductions were more evident in lung, stomach, and colorectal cancers, whereas differences were less pronounced for liver and pancreatobiliary cancers, particularly during earlier periods.

**Conclusion:**

These findings suggest that timely PC involvement may promote more judicious broad-spectrum antibiotic use in patients with advanced cancer.

**Disclosures:**

All Authors: No reported disclosures

